# Strategies for Identifying Patients for Deprescribing of Blood Pressure Medications in Routine Practice: An Evidence Review

**DOI:** 10.1007/s11906-024-01293-5

**Published:** 2024-02-02

**Authors:** James P. Sheppard, Athanase Benetos, Jonathan Bogaerts, Danijela Gnjidic, Richard J. McManus

**Affiliations:** 1 Nuffield Department of Primary Care Health Sciences, Radcliffe Primary Care Building, Radcliffe Observatory Quarter, University of Oxford, Oxford, OX2 6GG UK; 2 CHRU-Nancy, Pôle “Maladies du Vieillissement, Gérontologie Et Soins Palliatifs”, and Inserm DCAC u1116, Université de Lorraine, 54000 Nancy, France; 3 Department of Public Health and Primary Care, Leiden University Medical Center, Leiden, the Netherlands; 4 LUMC Center for Medicine for Older People, Leiden University Medical Center, Leiden, the Netherlands; 5 School of Pharmacy, Faculty of Medicine and Health, The University of Sydney, Sydney, NSW 2006 Australia

**Keywords:** Hypertension, Drug-related side effects and adverse reactions, Polypharmacy, Aging, Risk prediction, Frailty, Multi-morbidity

## Abstract

**Purpose of Review:**

To summarise the evidence regarding which patients might benefit from deprescribing antihypertensive medications.

**Recent Findings:**

Older patients with frailty, multi-morbidity and subsequent polypharmacy are at higher risk of adverse events from antihypertensive treatment, and therefore may benefit from antihypertensive deprescribing. It is possible to examine an individual’s risk of these adverse events, and use this to identify those people where the benefits of treatment may be outweighed by the harms. While such patients might be considered for deprescribing, the long-term effects of this treatment strategy remain unclear.

**Summary:**

Evidence now exists to support identification of those who are at risk of adverse events from antihypertensive treatment. These patients could be targeted for deprescribing interventions, although the long-term benefits and harms of this approach are unclear.

**Perspectives:**

Randomised controlled trials are still needed to examine the long-term effects of deprescribing in high-risk patients with frailty and multi-morbidity.

## Introduction

Medications that lower blood pressure have been studied for more than half a century, and it is well established that such treatment reduces the risk of stroke and cardiovascular disease across all age groups [1•]. As a result, antihypertensive medications are commonly prescribed [2], particularly in older adults, where more than half of individuals aged over 80 years receive therapy [3]. This has contributed to the steady decline in rates of cardiovascular disease seen globally over the past 50 years [4].

As with all medications, antihypertensive treatment is not without harm. Randomised controlled trials of blood pressure lowering therapy show that treatment is associated with an increased risk of hypotension, syncope, acute kidney injury and hyperkalaemia [5]. Overall, this risk is very low, affecting between 5 and 16 patients per 10,000 treated per year [6•]. However, in older patients and those with frailty, observational data suggest this risk is significantly increased, affecting up to 131 patients per 10,000 treated per year [6•]. This is thought to be because older people are more sensitive to the adverse effects of treatment, due to altered pharmacokinetic and pharmacodynamic responses [7], and because they are more likely to be prescribed multiple medications leading to polypharmacy [8], which increases the risk of drug-drug interactions leading to hospitalisation with adverse drug events such as serious falls.

In these patients, the benefits of antihypertensive treatment may eventually become outweighed by the harms and deprescribing has been proposed to reduce this risk [9]. However, identifying such patients is not straightforward and even when identified, clinicians may be unwilling to deprescribe. Current literature shows that treating clinicians feel comfortable to deprescribe antihypertensive treatment when prompted by a triggering adverse event or in context of terminal illness, but feel less confident doing this more proactively in presence of a limited life expectancy or general polypharmacy [10, 11]. The present review will summarise the current evidence for identifying patients who might benefit from deprescribing of blood pressure medications in routine practice.

## What is Deprescribing?

Deprescribing is a systematic process of discontinuing or reducing the use of medications that are considered inappropriate or unnecessary for a particular patient. An important element of deprescribing is that it is ‘supervised’ by a health care professional, typically a clinician or pharmacist, to manage polypharmacy and improve health outcomes [9]. Polypharmacy refers to the use of multiple medications by an individual, which can lead to various issues such as drug interactions, adverse effects, decreased medication adherence, and increased healthcare costs [12]. Deprescribing helps address these concerns by carefully evaluating the need for each medication and discontinuing those that are no longer beneficial or may pose risks.

At present, there is very little guidance on deprescribing antihypertensive medications [13•, 14]. Most clinical hypertension guidelines primarily focus on initiating and intensifying antihypertensive therapy [13•, 15, 16]. New guidelines from the European Society of Hypertension recognise for the first time the possibility for reducing antihypertensive treatment in older patients with frailty and low blood pressure (< 120 mm Hg), but they do not propose specific deprescribing strategies since it is acknowledged that these are not currently evidence based [13•]. As such, clinicians may have low confidence to implement deprescribing recommendations in everyday practice [17].

In contrast to drugs used for symptom control, antihypertensive medications are an appealing target for deprescribing since they are typically prescribed for prevention of cardiovascular disease and therefore may be stopped with very few adverse drug withdrawal events (i.e. most people who are prescribed antihypertensives will never experience a cardiovascular event) [18]. Deprescribing can be useful when continued antihypertensive treatment no longer aligns with the goals of care, particularly in situations such as end-of-life care, where the likelihood of obtaining further cardiovascular disease prevention benefits is minimal [19]. In such cases, deprescribing may be attempted due to the recognition of therapeutic futility.

Antihypertensive medications may also become inappropriate for patients who are at a high risk of experiencing adverse events. This can occur following a change in clinical status, for example, if a patient develops significant kidney impairment related to a particular medication class [20] or experiences a hypotension-related fall [21]. In these clearly defined cases, it may be necessary to discontinue or switch to an alternative medication. However, it may also be appropriate to reduce treatment in patients as they age and develop frailty when they become more susceptible to adverse events [6•]. In this situation, determining who is at high risk of adverse events can be challenging. Ideally, it requires a comprehensive assessment of the patient’s medical history, current health condition, medication regimen, and individual factors such as age, comorbidities, and functional status. However, this is rarely achieved in practice.

## Who are High-Risk Patients?

High-risk patients are those whose characteristics and medical history put them at greater risk of adverse events from antihypertensive therapy. These adverse events include hypotension, syncope, falls, fracture, acute kidney injury, and electrolyte abnormalities [5, 6•, 22]. Acute kidney injury and electrolyte abnormalities may often be attributable to specific antihypertensive drug classes [20, 23], meaning that it may be possible to switch patients to an alternative antihypertensive drug class to reduce their risk without the need for deprescribing.

**Fig. 1:**
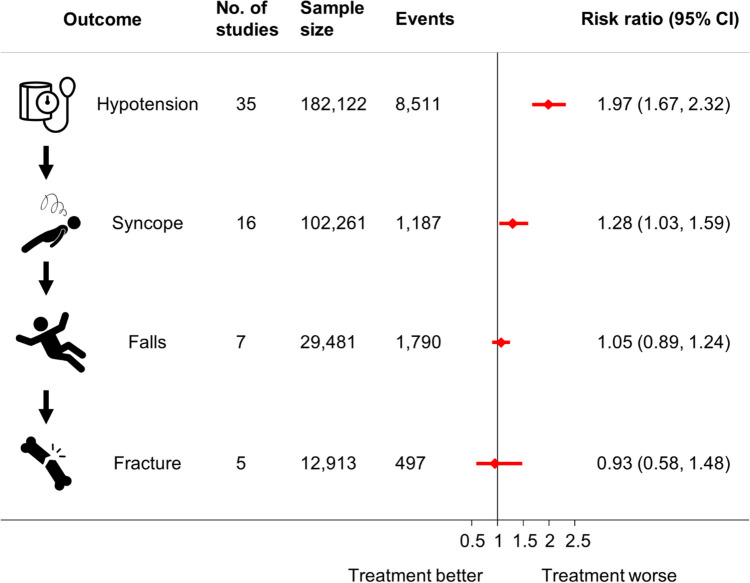
Association between antihypertensive treatment and adverse events. Data from the meta-analysis of randomised controlled trials by Albasri et al. (2021) [5]. CI, confidence intervals

Other common adverse events that line up along the same causal pathway (Fig. 1) and occur as a result of blood pressure lowering itself may be a trigger for deprescribing. In this scenario, events more closely related to the process of blood pressure lowering (e.g. hypotension and syncope) have been shown to have a stronger relative association with antihypertensive treatment in randomised controlled trials (Fig. 1) [5]. Those events further along the causal pathway from treatment appear to have weaker associations [5]; however, these may be considered to be more serious, resulting in hospitalisation and even death [6•]. As a result, even small relative effects may be important, particularly if an individual’s underlying risk of harm is high.

There are many conditions and factors that could lead someone to have a higher underlying risk of adverse events from antihypertensive treatment (Table 1). Many are related to advancing age, medical history (including dementia), and medication prescriptions, which are generally straightforward to assess in routine clinical practice. However, using these factors alone to determine eligibility for deprescribing can be overly simplistic and may not capture the full complexity of an individual’s health status and treatment needs. Indeed, consideration of blood pressure and cardiovascular risk may also be important since, for some patients with many risk factors, continued treatment may still be appropriate and beneficial [13•].

**Table 1:** Examples of patients at high risk of harm from antihypertensive treatment who may benefit from deprescribing interventions

**Risk factor**	**Description**
**Advancing age**	As individuals age, their body’s ability to process medications may change, making them more susceptible to adverse effects. They may also have multiple chronic conditions and take numerous medications, increasing the potential for drug interactions and adverse events.
**Dementia**	People with dementia are at an increased risk of adverse events such as syncope and falls. Certain medications commonly used in patients with dementia, such as sedatives or antipsychotics, can further contribute to these risks and may be a greater priority for deprescribing or adjustment. In addition, anti-cholinesterase medications proposed in Alzheimer diseases can be responsible for bradycardia and conduction disturbances especially when prescribed in association with beta-blockers.
**Chronic kidney disease**	Chronic kidney disease can affect the clearance of medications from the body, leading to a higher risk of drug accumulation and toxicity, which in turn can increase the risk of adverse events such as acute kidney injury.
**History of adverse events**	Individuals who have experienced adverse events related to specific medications in the past may be considered high risk of further complications. Deprescribing those medications or finding suitable alternatives can reduce the likelihood of recurring adverse events.
**Low blood pressure**	Patients with low systolic blood pressure (< 120 mm Hg) are at great risk of hypoperfusion and syncope-related adverse events and may be able to tolerate antihypertensive deprescribing better than individuals with higher blood pressures.
**Polypharmacy**	Polypharmacy increases the risk of drug-drug interactions, side effects, and medication errors. Sometimes, polypharmacy may be entirely appropriate, given the number of conditions present. In this situation, although patients are at increased risk of hospital admission due to adverse drug events, the benefits of polypharmacy may still outweigh the risks. However, in patients with *inappropriate* polypharmacy, the benefits of treatment may be outweighed by the harms. Thus, the role of the clinician (ideally in collaboration with a clinical pharmacist) should be to establish the benefit/risk ratio of each drug in a given patient and then to prioritise the therapeutic indications in order to reduce polypharmacy as much as possible.
**Severe frailty**	Frailty refers to a state of increased vulnerability and decreased physiological reserve. Frail individuals are more prone to serious adverse medication effects, such as falls, which can result in hospitalisation and reduced independence in this population.
**Combination of risk factors**	Some patients may have a combination of risk factors, such as older age, multiple chronic conditions, impaired organ function, and polypharmacy. These individuals are particularly susceptible to adverse events, but in routine clinical practice, it is difficult to identify such patients in a systematic manner.

Another important factor to consider when identifying potential candidates for deprescribing is clinical frailty [24]. Frailty is a multidimensional concept that encompasses physical, cognitive, and social aspects. Frail individuals often have decreased physiological reserve, altered pharmacokinetics, and increased susceptibility to adverse drug reactions [24]. Ideally, healthcare professionals should take into account a person’s level of frailty, along with other clinical factors, when evaluating the appropriateness of medication use [25] and the potential benefits of deprescribing [13•, 26].

## Measuring Frailty

There are various frailty assessment tools available, but their implementation and interpretation can vary in clinical practice. Comprehensive geriatric assessments can provide a more holistic understanding of a patient’s risk profile, enabling more targeted deprescribing. Commonly used approaches to measuring frailty include the Clinical Frailty Scale [27], frailty phenotype models [28], and the cumulative deficit model [29].

### The Clinical Frailty Scale

The Clinical Frailty Scale [27] is a widely used tool to assess frailty in clinical settings, particularly since the COVID-19 pandemic when it was widely used to allocate limited resources such as beds in intensive care units [30]. It is a visual rating scale that provides a holistic assessment of a person’s overall health and functional status. The scale ranges from 1 (very fit) to 9 (terminally ill), with different levels representing different degrees of frailty [27]. It is very easy to use but highly subjective, requiring a healthcare professional to evaluate the patient based on their physical and cognitive abilities and functional independence to determine their level of frailty [27].

### Phenotype Model

Specific frailty phenotypes are assessed using questionnaires or specific assessments such as the timed-up-and-go test and hand-grip strength test. Questionnaires’ assessments based on phenotypes of frailty typically involve a series of questions related to physical functioning, mobility, activities of daily living, and other relevant factors. Common questionnaires include the Fried Frailty Phenotype [28], Edmonton Frail Scale [31], and the Tilburg Frailty Indicator [32]. Each questionnaire needs to be either administered by a healthcare professional or via self-report, and frailty categories are determined by various scoring systems based on the answers provided.

### Cumulative Deficit Model

The Cumulative Deficit Model evaluates frailty based on the accumulation of deficits in various domains related to physical, cognitive, psychological, and social factors [29]. Referred to as a ‘frailty index’, it involves assessing the presence or absence of specific deficits or impairments, such as chronic diseases, mobility limitations, sensory impairments, and cognitive decline. The total number of deficits present is divided by the total number of deficits considered to give a value of between 0 and 1, with higher values indicating more severe frailty [29]. This approach has become increasingly popular due to its reproducibility and ease of administration, with newer versions developed for integration into routine electronic health record systems [33].

Regarding the level of frailty at which deprescribing should be considered, there is currently no universally agreed-upon threshold. The general concept is that deprescribing should be considered in patients with severe frailty and loss of autonomy in order to reduce adverse effects related to both polypharmacy and very low blood pressure [26]. However, like many other risk factors, those with more severe frailty are also more likely to have compelling cardiovascular conditions and higher risks for cardiovascular complications [34] and therefore benefit from continued antihypertensive therapy. As a result, the appropriateness of deprescribing based on frailty should be assessed on a case-by-case basis, considering the potential benefits, risks, and individualised goals of care. Regular monitoring and reassessment of frailty status are essential to ensure that deprescribing decisions remain appropriate and aligned with the individual’s changing health condition.

## Measuring the Risk of Adverse Events

Given the complexity of hypertension management in older patients with frailty and multi-morbidity, it may be appropriate to examine an individual’s risk of specific adverse events by considering a variety of factors and then weigh this against their likelihood of benefiting from continued treatment. For this, one can draw a parallel with the management of anticoagulation treatment in patients with atrial fibrillation, where the CHA_2_DS_2_-VASc and HAS-BLED scores can be used to assess the likelihood of benefit (in terms of reducing the risk of stroke) versus harm (by increasing the risk of a serious bleed) [35]. Whilst using these tools to estimate an individual’s risk of cardiovascular disease are commonplace (e.g. QRISK3 [36], SCORE [37]), equivalent tools to estimate the risk of adverse events related to antihypertensive therapy have only recently been developed.

The STRATIFY-Falls tool uses information routinely available in primary care electronic health records to estimate an individual’s risk of going to hospital or dying from a serious fall [38•]. The model includes various risk factors, such as age, sex, ethnicity, history of falls, stroke, and multiple sclerosis, frailty (determined by the electronic frailty index) [33], and medication use (including antihypertensive medication prescription) to calculate the likelihood of a serious fall within the next 1, 5, and 10 years [38•]. The model has been externally validated, showing excellent discrimination and good calibration in most patients, except those at very high risk [38•]. Similarly, the STRATIFY-AKI tool uses commonly available information within an individual’s electronic health record to estimate the risk of hospitalisation or death from acute kidney injury within the next 1, 5, or 10 years [39]. Upon external validation, this model also showed excellent discrimination and reasonable calibration across the vast majority of patients attending primary care, potentially eligible for blood pressure lowering treatment [39].

These models have advantages in that they can be integrated into electronic health record systems to provide decision support for clinicians considering deprescribing of antihypertensive medications. They provide personalised risk estimates, which can be directly weighed against cardiovascular risk estimates to identify patients at high risk of harm but low risk of benefit. However, they also have limitations, such as variation in predictive accuracy across different populations, particularly those who are at very high risk of adverse events [38•]. Also, when considering the risks generated by these models, there is uncertainty regarding the threshold of risk that should be considered high enough to warrant deprescribing. Such a threshold is likely to vary from person to person, and therefore, it is crucial to engage in shared decision-making between healthcare professionals and patients [40]. This would include consideration of their values, preferences, and the individualised assessment of benefits and risks [41]. However, very old adults often choose to rely entirely on their doctor regarding the choice of treatments, which does not foster a relationship that is conducive to shared and informed decision-making [42]. It is argued that changing this relationship represents a fundamental challenge to deprescribing in routine clinical practice [42].

## Understanding the Potential Benefits and Harms of Treatment

**Fig. 2:**
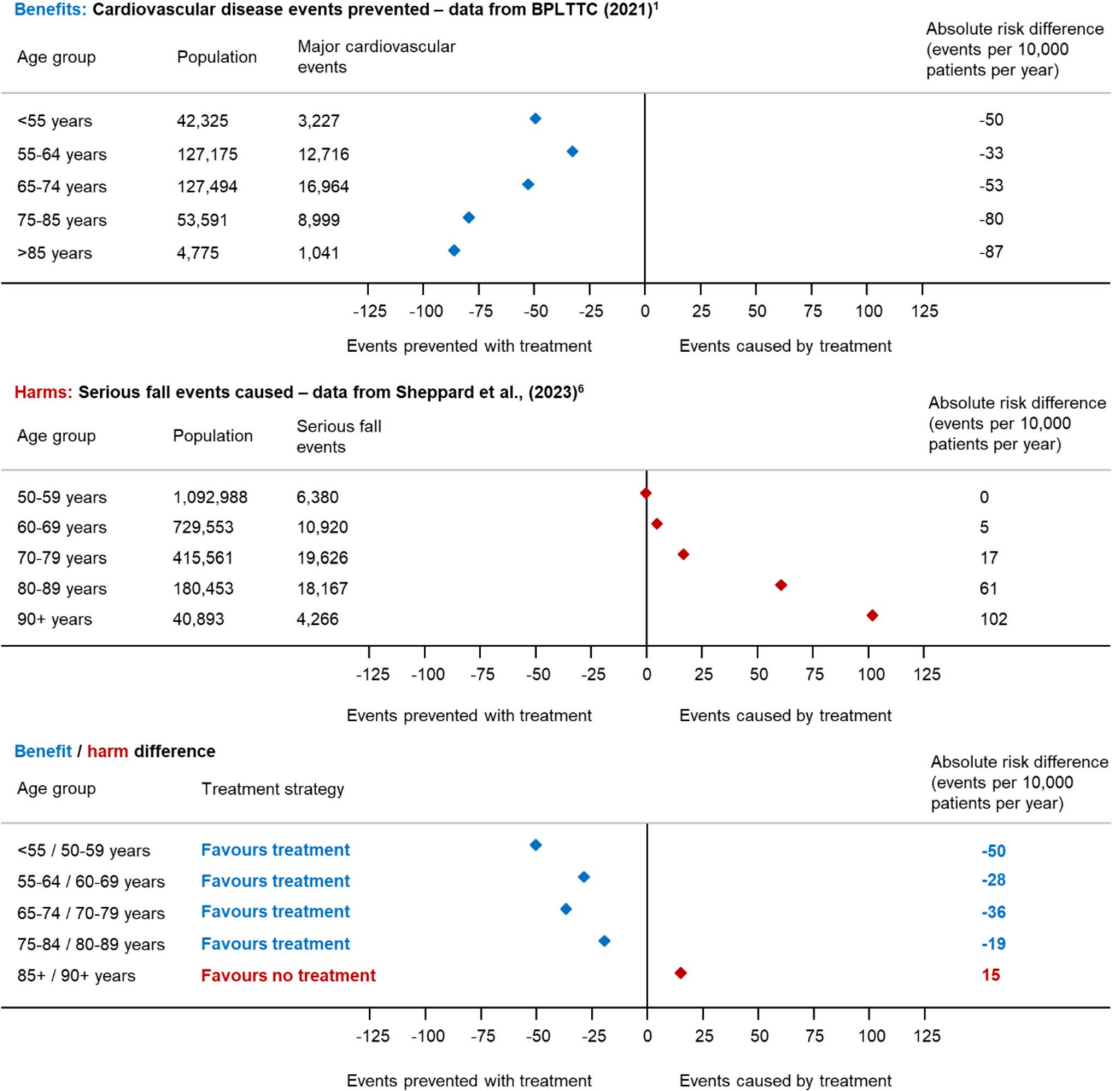
Changes in the benefits and harms of treatment by age. This figure compares the absolute risk of cardiovascular disease events that are reduced by antihypertensive treatment (using data from randomised controlled trials in the Blood Pressure Lowering Treatment Trialist’s Collaboration [BPLTTC]) [1•] with the absolute risk of serious falls which are increased by antihypertensive treatment (using observational data from routine electronic health records) [6•]

It is also important to weigh an individual’s likelihood of benefit from treatment against their likelihood of harm when considering potential patients for deprescribing. This should not just take into account the relative effect of treatment on a given outcome, but combine this with the underlying risk of the individual concerned. This is illustrated in Fig. 2, where the absolute risk of cardiovascular disease with treatment is compared to the absolute risk of serious falls with treatment prescription, using data from contemporary clinical trials and observational studies [1•, 6•]. This shows that in younger patients in their 50 s and 60 s, the absolute risk reduction in cardiovascular disease with treatment far outweighs the increased risk of a serious fall, with 28–50 more cardiovascular events prevented than serious falls caused per 10,000 patients treated per year. However, as patients approach older age, the absolute risk of serious falls with treatment increases, meaning that for a 90-year-old, antihypertensive treatment is more likely to cause a serious fall, than prevent a cardiovascular event (15 more serious falls caused than cardiovascular disease events prevented per 10,000 patients treated per year).

In this illustration, age alone is used as a way of determining how the underlying risk of an individual affects the ratio of benefit to harm with antihypertensive treatment. However, in routine clinical practice, understanding this balance is much more complicated. When considering deprescribing, it is important to remember that most antihypertensive drugs are also indicated for the most frequent and serious age-related cardiovascular diseases (e.g. heart failure, atrial fibrillation, coronary heart disease), and therefore, it is essential to have a good understanding of the existence of such diseases in a given patient before modifying treatment. In very old and frail subjects, this can be difficult, since the symptoms of these diseases (fatigue, dyspnea, mobility disorders, peripheral edema, etc.) are multifactorial and the contribution of the cardiovascular system is not easy to assess for non-experts. It is therefore important that deprescribing decisions are made in collaboration between the primary care clinician, geriatrician, cardiologist and pharmacist, using as much information about an individual’s health status and underlying risk as possible. Here, the STRATIFY tools [38•, 39] described above may be useful in providing insights as to which patients are most likely to be at non-cardiovascular-related harm.

## How to Deprescribe Antihypertensive Medication

**Fig. 3:**
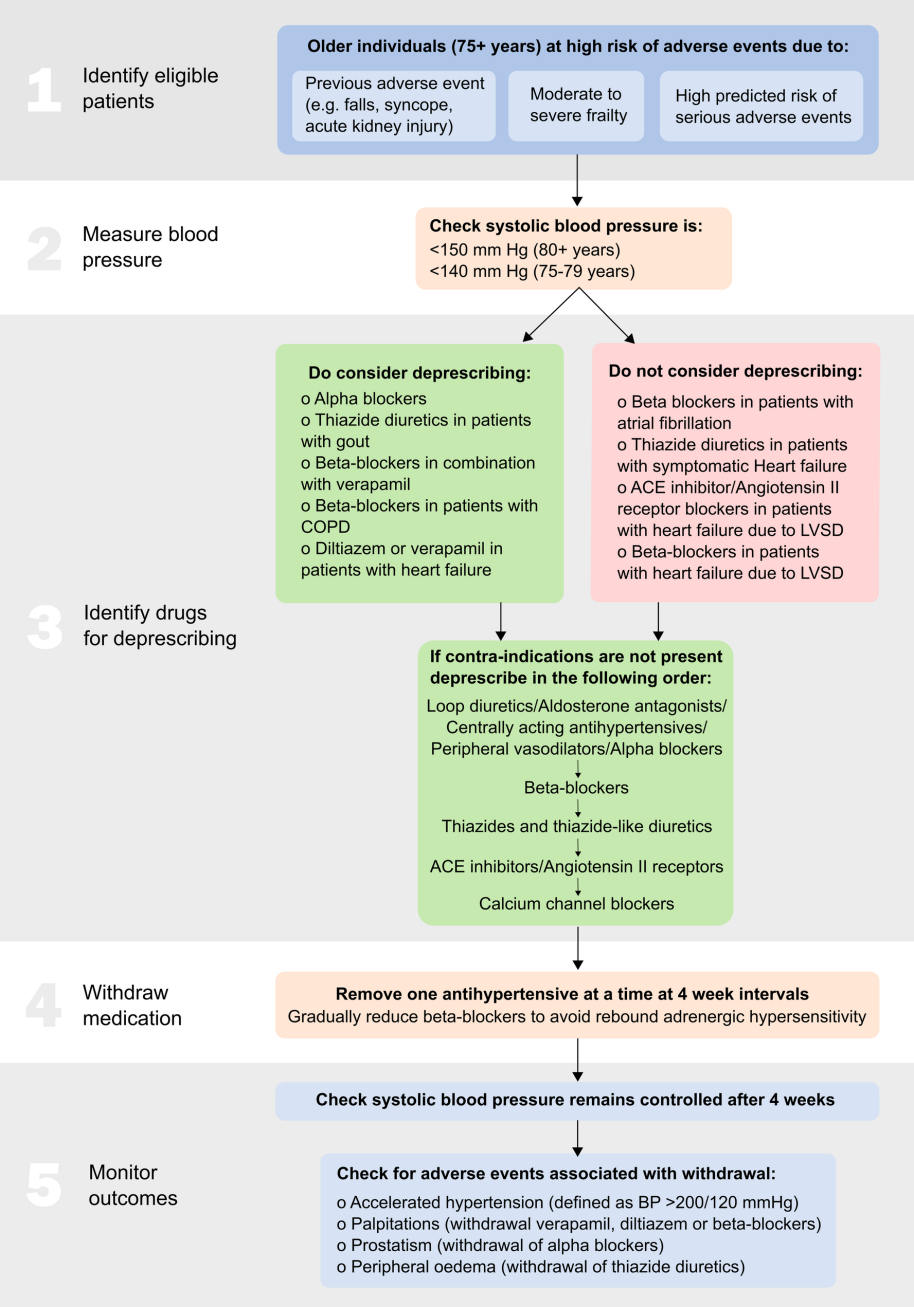
Antihypertensive deprescribing algorithm. ACE, angiotensin-converting enzyme; LVSD, left ventricular systolic dysfunction; COPD, chronic obstructive pulmonary disease; BP, blood pressure. [2022] Sheppard et al. Reprinted with permission from [43]

Deprescribing should only be attempted by an appropriately qualified medical professional, which for most patients with uncomplicated hypertension will be their primary care clinician, nurse or a pharmacist with prescribing qualifications. In those high-risk patients where a decision has been made to deprescribe antihypertensive therapy, these healthcare professionals may wish to consider the following key steps (summarised in Fig. 3) [43].

### Measure Blood Pressure

Before withdrawing treatment, it is important to ensure that the patient’s blood pressure is controlled below the recommended levels according to clinical guidelines [13•, 15, 16]. Typically, for patients aged 80 years or older, the clinic systolic blood pressure should be below 150 mm Hg, while for younger patients, it should be below 140 mm Hg [16]. Deprescribing is more likely to be successful in patients with lower systolic blood pressure, such as readings below 120 mm Hg [13•]. However, in cases of life-limiting illness, the threshold for intervention may differ due to the futility of treatment, except at the highest levels of blood pressure [44].

### Identify Drugs for Deprescribing

To identify candidate drugs for deprescribing, a thorough review of the patient’s current medication regimen is required. Tools such as STOPP/START [45•], STOPPFrail2 [46], and the American Geriatrics Society Beers’ criteria [47] can be used to identify contraindications for antihypertensive medications that may have arisen due to concomitant prescriptions or newly developed conditions. In some cases, it may be inappropriate to deprescribe antihypertensive medications, especially if they have been prescribed for indications other than blood pressure management. Examples include beta-blockers prescribed for atrial fibrillation, diuretics in patients with symptomatic heart failure, and ACE inhibitors/angiotensin II receptor blockers/aldosterone antagonists or beta-blockers in patients with heart failure and reduced left ventricular ejection fraction.

When there are no contraindications, healthcare professionals should identify all prescribed antihypertensive medications and withdraw them one by one, following a reverse of guideline-recommended treatment [13•, 15, 16]. For example, medications that are not recommended for older adults, such as loop diuretics, aldosterone antagonists, centrally acting antihypertensives, peripheral vasodilators, and alpha-blockers, could be the first to be stopped. Among commonly used drugs, medications could be stopped according to effectiveness in the older population, with beta-blockers being considered for withdrawal first, followed by thiazide and thiazide-like diuretics or ACE inhibitors/angiotensin II receptor blockers, and finally, calcium channel blockers [48].

### Tailor Down-titration of Medications According to Drug Class

Deprescribing antihypertensive medications should be done gradually and with close monitoring. If withdrawing beta-blockers, reducing the dose before a complete withdrawal is advisable to avoid rebound adrenergic hypersensitivity. A progressive strategy can also be applied for diuretics, especially in patients on high doses of loop diuretics, to mitigate the risk of salt/water retention.

### Monitor Outcomes Carefully

During the process of deprescribing, antihypertensives can be withdrawn one at a time at 4-week intervals [49•]. Regular follow-ups and blood pressure measurements can help guide the deprescribing process and ensure that blood pressure remains within an acceptable range (e.g. < 140 mm Hg in patients aged below 80 years, < 150 mm Hg in patients aged 80 years and older) [16]. If blood pressure becomes uncontrolled, the healthcare professional may consider reintroducing the previously withdrawn medication at a lower dose, if available, or explore non-pharmacological approaches to blood pressure reduction [50].

An important concern for patients and healthcare professionals considering deprescribing of antihypertensive treatment is what will happen when medications are stopped. There is also a need for more resources to support patients in whom deprescribing of antihypertensives is recommended [51]. However, currently there is limited evidence available to support clinicians in routine practice, with just a few short-term trials examining this treatment strategy.

One recent trial, the OPtimising Treatment for MIld Systolic hypertension in the Elderly (OPTiMISE) trial [49•], examined the short-term safety and efficacy of antihypertensive deprescribing. This trial focused on withdrawing one antihypertensive medication in patients aged 80 years or older with baseline systolic blood pressure below 150 mm Hg who were prescribed two or more antihypertensive medications. Among the 569 participants, all of those randomised to the intervention group successfully deprescribed their therapy, and 66% maintained this medication reduction throughout the 12-week follow-up period. The trial did not find any significant difference in the proportion of patients with controlled blood pressure at follow-up, and there were no noticeable differences in serious adverse events leading to hospitalisation or death. However, it is important to note that the number of events was low (10 in the control group versus 13 in the intervention group). Furthermore, a recent systematic review published in the Cochrane Library [52•] analysed all available evidence on antihypertensive deprescribing based on randomised controlled trials (not including the OPTiMISE trial [49•], which was published later) and identified six trials with a total of 1073 participants. Due to the low number of outcome events, the analysis found no significant associations between antihypertensive deprescribing and all-cause mortality (four studies, 18 outcome events), myocardial infarction (two studies, three events), stroke (three studies, five events), and all-cause hospitalisation (one study, 19 outcome events) [52•].

Even more recently, a small study [53] assessed the feasibility of deprescribing antihypertensive treatment in adults aged 75 years or older with two or more antihypertensive drugs that had a physical complaint mentioned in their electronic patient record related to these drugs. During a 1-year follow-up period, 11 out of 14 (79%) participants deprescribed a portion of their antihypertensive treatment while their blood pressure was maintained at an acceptable level, and 9 out of 14 (64%) reported no further adverse drug events after 12 months [53]. Finally, a trial not yet published in full on antihypertensive deprescribing in nursing home residents with dementia was stopped early on advice of the Data Safety Monitoring Board [54].

At present, these findings do not provide sufficient evidence to determine whether or not antihypertensive deprescribing should be attempted in older patients with frailty. While evidence specific to antihypertensive deprescribing is limited, healthcare professionals can utilise clinical judgment, patient preferences, and ongoing monitoring to guide the process.

## Implications for Clinical Practice

Patients are likely to prioritise the benefits and harms of antihypertensive treatment differently based on their values, preferences, and specific circumstances [55]. Clinical practice should involve shared decision-making, where healthcare professionals engage patients and their caregivers in discussions about the risks and benefits of different treatment options, including deprescribing, although this has been noted as a challenge for deprescribing in practice [42]. The ultimate goal should be to optimise patient care by weighing the benefits and harms of continued treatment in light of each patient’s unique circumstances. Aiding healthcare professionals, patients, and their caregivers with accurate and comprehensive information about the risks and benefits associated with treatment is crucial. This is particularly important, given the limited evidence regarding long-term outcomes of deprescribing: healthcare professionals should therefore acknowledge that deprescribing, particularly in the context of antihypertensive medications, is an area with limited evidence, with very few clinical trials assessing long-term clinical effects [49•, 52•]. While identifying high-risk patients may suggest deprescribing as a potential strategy, it does not guarantee that it is the optimal treatment approach for every patient.

## Conclusions

When the benefits of antihypertensive treatment may eventually become outweighed by the harms, deprescribing antihypertensive medication represents a potential strategy to address polypharmacy in older patients with increasing frailty. Challenges exist in the identification of high-risk patients who might benefit from such an intervention and the understanding of the process of deprescribing itself in routine clinical practice. Factors such as age, blood pressure, medical history, medication prescriptions, and frailty can influence the decision-making process, but at present, there are limitations in our understanding of how to best examine frailty and assess the risk of specific adverse events in routine practice. As a result, decisions about deprescribing should ideally be made in collaboration with the primary care clinician, geriatrician, cardiologist and pharmacist. The outcomes of deprescribing remain uncertain, and few clinical trials have assessed the long-term clinical effects. While deprescribing may be appropriate for some patients, it may not always be the optimal strategy. Close monitoring, regular reassessment, and ongoing communication are essential to navigate the complexities of deprescribing and to provide personalised care that aligns with each patient’s goals and needs.
